# Additive Manufacturing of Bio-Based PA11 Composites with Recycled Short Carbon Fibers: Stiffness–Strength Characterization

**DOI:** 10.3390/polym17182549

**Published:** 2025-09-20

**Authors:** Christian Brauner, Thierry Bourquin, Julian Kupski, Lucian Zweifel, Mohammad Hajikazemi, Chester Houwink, Martin Eichenhofer

**Affiliations:** 1 Institute of Polymer Engineering, FHNW University of Applied Sciences and Arts Northwestern Switzerland, Klosterzelgstrasse 2, 5210 Windisch, Switzerland; thierry.bourquin@students.fhnw.ch (T.B.); julian.kupski@fhnw.ch (J.K.); lucian.zweifel@fhnw.ch (L.Z.); mohammad.hajikazemi@fhnw.ch (M.H.); 2 9T Labs AG, Badenerstrasse 790, 8048 Zürich, Switzerland; chester@9tlabs.com (C.H.); martin@9tlabs.com (M.E.)

**Keywords:** additive fusion technology, short carbon fiber, polyamide 11, bio-based polymer, recycled carbon fiber, 3D printing, injection molding, fiber orientation, mechanical properties

## Abstract

Short carbon fiber-reinforced bio-based polyamide 11 (PA11) composites were developed in filament form for Additive Fusion Technology (AFT) 3D printing and benchmarked against injection-molded samples. Composites containing 15 and 25 weight percent (wt%) recycled carbon fibers (rCFs) were successfully extruded into 1.75 mm diameter filaments, whereas higher fiber contents (35 wt%) led to brittle filament failure. AFT printing with subsequent consolidation produced short fiber composites with highly aligned fibers, while injection molding generated more randomly oriented microstructures. Mechanical testing revealed that AFT-printed composites in the fiber direction achieved significantly higher stiffness and comparable tensile strength to injection-molded counterparts. At 25 wt% fiber content, AFT 0° specimens reached an axial tensile modulus of 14.5 GPa, about 32% higher than injection-molded samples (11.0 GPa), with similar axial tensile strength (~123 vs. 126 MPa). However, AFT specimens displayed pronounced anisotropy: transverse (90°) properties dropped to ~2.3 GPa for transverse modulus and ~46–50 MPa transverse tensile strength, near matrix-dominated levels. Impact testing showed orientation-dependent toughness, with AFT 90° samples at 15% fiber content achieving the highest impact energy (76 kJ·m^−2^), while AFT 0° samples were ~30% lower than injection-molded equivalents. Dynamic mechanical analysis confirmed that AFT 0° composites maintained higher stiffness up to ~80 °C. Overall, these results demonstrate that aligned short fiber filaments enable high stiffness and strength performance comparable to injection molding, with the trade-off of anisotropy that must be carefully considered in design. This study is the first to demonstrate the feasibility of combining bio-based PA11 with recycled short carbon fibers in AFT printing, thereby extending additive manufacturing to sustainable and high-stiffness short fiber composites.

## 1. Introduction

Fiber-reinforced composites are essential in lightweight structural applications across many sectors such as aerospace, automotive, and energy. Their high strength-to-weight ratio enables significant performance gains, but sustainability concerns have intensified interest in bio-based matrices and recycled fiber reinforcements [1]. Polyamide 11 (PA11), a fully bio-based polymer derived from castor oil, combines low moisture absorption with high toughness compared to conventional nylons (e.g., PA6, PA12), making it an attractive sustainable matrix for advanced composites [2]. When combined with recycled carbon fibers (rCFs), PA11-based composites offer the potential to reduce both environmental impact and material costs, while retaining much of the performance of virgin carbon fiber systems.

The growing use of carbon fiber-reinforced polymers (CFRPs) has generated increasing volumes of manufacturing scrap and end-of-life waste. Landfilling and incineration of CFRPs are no longer acceptable from regulatory and environmental perspectives, driving the development of recycling technologies for CF recovery [3,4,5]. Three main routes dominate: (i) mechanical recycling (milling, shredding), which is inexpensive but produces short, damaged fibers of limited reinforcing potential [6]; (ii) thermal recycling (pyrolysis, fluidized bed, microwave), which decomposes the matrix and recovers fibers with 80–95% of virgin tensile strength, albeit with surface oxidation and high energy demand [7,8]; and (iii) chemical recycling (solvolysis), which dissolves or depolymerizes resins under sub- or supercritical conditions, yielding near-virgin-quality fibers and sometimes resin oligomers [9]. Recent works have also explored greener recycling processes, such as subcritical water treatments [10] or based on the glass transition principle [11], to reduce environmental impact.

Recycled carbon fibers are generally discontinuous, making them suitable for short-fiber composites. In thermoplastics, injection-molded rCF composites show nearly linear increases in stiffness and strength with fiber content [12], although poor interfacial bonding often limits impact resistance [13]. In thermosets, rCFs have been integrated into sheet- and bulk-molding compounds (SMC/BMC), partially replacing glass fibers in industrial production [14]. Nonwoven mats of rCF [15] can be compression-molded into samples with quasi-isotropic properties [16]. More recently, rCF has been compounded into thermoplastic filaments for additive manufacturing. In fused filament fabrication (FFF), nozzle shear aligns short fibers along deposition paths, resulting in high in-plane stiffness, but anisotropy and porosity remain challenges [17].

While rCF generally retains most of its stiffness, tensile strength is often reduced by 10–30% due to surface defects and loss of sizing during recycling [8,18]. This weakens fiber–matrix adhesion, though re-sizing and surface treatments can restore interfacial properties [19,20,21]. Fiber attrition during compounding further reduces aspect ratios, limiting reinforcement efficiency. Therefore, processing methods that enhance fiber alignment (e.g., injection molding flow, print path design) are critical in achieving the required stiffness and strength properties [17,22]. Encouragingly, bio-based matrices such as PLA have been reinforced with rCF, demonstrating the feasibility of fully sustainable composites [23,24].

Traditional manufacturing of short fiber composites is dominated by injection molding, which can efficiently produce complex parts with fiber loadings up to ~50 wt% [25]. In injection-molded parts, fibers tend to align with flow near the mold walls but are more randomly oriented in the core [26,27] in all three directions, leading to a moderate overall fiber orientation. Consequently, injection-molded short fiber composites with a large core and shell effect often exhibit relatively isotropic properties and high toughness, but suboptimal stiffness and strength compared to continuous fiber composites [28]. The influence of fiber orientation in injection-molded samples complicates the analysis [29] and, consequently, the design of short-fiber composites, as their behavior differs significantly from that of continuous carbon-fiber composites. This leads to a severe plastic response, accompanied by multiple microscopic damage events, even in the longitudinal direction parallel to the main flow [29].

In contrast, additive manufacturing techniques now enable greater control over fiber orientation. Additive Fusion Technology (AFT), developed by 9T Labs [30,31], is a fused filament fabrication (FFF) based process tailored for carbon fiber-reinforced thermoplastics. In AFT, short filaments or endless tape like filaments can be laid down in tailormade orientations. For the short fiber filaments, the shear flow through the nozzle highly aligns the short fibers (typically less than 0.2 mm, smaller than the printing nozzle 0.4–0.8 mm) along the extrusion direction. By changing the nozzle diameter, the alignment of the fibers can be directly influenced. This can produce parts with nearly unidirectional fiber alignment in each layer without changes over the thickness of the part, analogous to an automated fiber placement approach for continuous fibers. A subsequent consolidation step (typically hot pressing the printed preform) is employed to eliminate voids and merge the filament interfaces, which reduces porosity from potentially 10–30% in the as-printed state to below 1%, thereby restoring composite performance. The combination of highly oriented fiber architecture and low void content achieved by AFT promises can significantly enhance stiffness and strength along the print direction, while using short (and even recycled) fibers. Increasing fiber alignment in short fiber composites correlates with higher tensile strength but lower ductility. The trade-off is that strongly aligned microstructures can exhibit poor transverse properties and reduced fracture toughness. Given these considerations, it is crucial to compare AFT-printed short fiber composites against their conventional injection-molded counterpart to evaluate the benefits and drawbacks [32]. While several studies have explored rCF-based composites for injection molding and conventional FFF printing, the potential of highly aligned rCF-PA11 composites processed via AFT remains largely unexplored [30].

In this work, we focus on short carbon fiber-reinforced PA11 composites with an emphasis on sustainability (bio-based polymer matrix and recycled fiber use). A 1.75 mm diameter filament with various fiber loadings has been developed for AFT printing, and the practical fiber content limit for filament extrusion has been determined. The material characterization includes fiber length distribution after compounding, microstructural analysis of fiber orientation, and mechanical testing (tensile properties, Charpy impact toughness, and dynamic mechanical analysis). Two different manufacturing routes for the same material system have been used: (1) AFT (FFF printing + consolidation) plates that are machined into test specimens with fibers primarily aligned (0°) or transverse (90°) to the loading direction; and (2) injection molding of standardized specimens, which serves as a baseline with a more random fiber orientation distribution. By comparing these, the influence of fiber orientation and processing on composite performance is elucidated. The results are discussed in the context of fiber attrition during processing and the known relationships from composite theory. This study provides insights into the design of sustainable short fiber composites for additive manufacturing and identifies the current limitations, such as fiber content thresholds and anisotropy, that must be addressed to fully capitalize on recycled fibers in 3D-printed high-performance parts.

Although recycled carbon fibers (rCFs) have been successfully integrated into thermoplastic composites by conventional methods such as injection molding or compression molding, limited research has investigated their use in filament-based additive manufacturing processes, particularly in combination with bio-based polymer matrices. Most prior studies on additive manufacturing with rCF have focused on polylactide (PLA) or petroleum-based nylons, while the processing limits, fiber alignment effects, and performance trade-offs of bio-based PA11/rCF composites remain unexplored. Furthermore, the specific potential of Additive Fusion Technology (AFT) to produce highly aligned short-fiber composites from recycled feedstock has not been systematically evaluated against established processes like injection molding.

The present work addresses this gap by developing rCF-reinforced PA11 filaments suitable for AFT printing and benchmarking their mechanical and thermal performance against injection-molded counterparts. The study defines practical processing limits in terms of fiber content, analyzes fiber length attrition and orientation, and quantifies stiffness, strength, impact toughness, and temperature-dependent properties. By performing so, it establishes both the opportunities and limitations of AFT for sustainable short-fiber composites and outlines design considerations for future structural applications.

## 2. Materials and Methods

### 2.1. Fiber and Matrix Materials

Polyamide 11 (PA11): A commercial 100% bio-based PA11 (Rilsan^®^ Arkema, Puteaux, France) was used as the polymer matrix. This grade is characterized by low moisture uptake and excellent toughness relative to other polyamides. The neat PA11 has a tensile strength of ~37 MPa and elongation > 50% according to the datasheet. Prior to processing, the PA11 pellets were dried at 80 °C for >4 h to remove moisture.

Carbon Fibers: Although the project objective was to evaluate recycled carbon fibers, virgin T700-grade fibers (Suter Kunststoffe AG, Fraubrunnen, Switzerland) were used as a controlled baseline to ensure consistent quality during process development; these were provided unsized. To simulate the condition of recycled fibers (which often lack sizing after reclamation), the fibers were deliberately used without the original sizing and then re-sized with compatible treatment. This would be similar to a pyrolysis provided by companies like MCAM (former CarboNXT, Gainesville, FL, USA), Composite Recycling or solvolysis process related to V-Carbon to reclaim the fibers.

#### Limitations Related to Recycled Fibers

Surface Chemistry: Recycled carbon fibers often lose their original sizing during pyrolysis or solvolysis. This leads to surface oxidation, roughness, or residual contaminants that weaken fiber–matrix adhesion. Re-sizing treatments are needed to restore compatibility with PA11, otherwise interfacial strength and toughness decrease.

Diameter Distribution: Unlike virgin fibers, which have tightly controlled diameters, recycled fibers can contain a mixture of high-tensile and high-modulus grades. This variability introduces inconsistency in load transfer and mechanical performance.

Residues and Impurities: Depending on the recycling route (mechanical, thermal, chemical), recycled fibers can contain resin residues, char, or dust-like fragments. These reduce the effective aspect ratio, contribute to porosity, and lower reinforcement efficiency.

Fiber Length Attrition: During compounding, recycled fibers suffer further breakage, often reducing 6 mm input fibers to average lengths of ~0.1 mm. Higher loadings accelerate this attrition, diminishing reinforcement efficiency and setting a practical upper limit near 25–30 wt%.

A polyurethane-based sizing (Michelman Hydrosize^®^ U502, Cincinnati, OH, USA) was applied to the fibers (approximately 1:10 in deionized water) to improve interfacial bonding with PA11. The fiber supplier’s lot contained a mix of >90% high-tensile-strength fibers and <10% high-modulus fibers, reflecting typical rCF variability.

### 2.2. Composite Preparation

**Figure 1:**
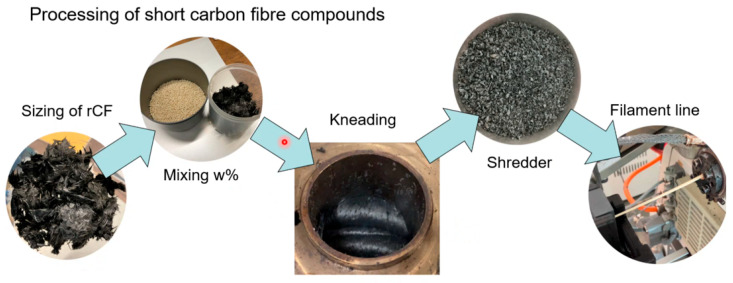
Processing of short carbon fiber compounds.

Compounding: Three fiber weight fractions (15 wt%, 25 wt%, 35 wt%) were targeted for the PA11/CF compounds. Compounding was performed in batch mode using a laboratory kneader (HAAKE™ Rheomix OS Lab Mixer, Thermo Fischer, Basel, Switzerland) at 230 °C for ~15 min per batch. A kneader was used to ensure a constant weight fraction compared to twin-screw extrusion. In each batch, ~200 g of PA11 pellets were first melted and brought to processing temperature, then the pre-weighed carbon fibers were gradually added over the course of mixing. A gradual fiber addition was necessary to avoid over-torque and ensure homogeneous dispersion. The fibers were added only after the polymer was fully molten, minimizing fiber residence time at high shear to reduce excessive breakage. Even so, significant fiber length reduction during compounding was expected, especially at the higher fiber loadings due to increased fiber–fiber interactions. After mixing, the compound was discharged, cooled, and granulated into ~3 mm pellets (Figure 1).

Filament Extrusion: The compounded pellets were then extruded into 1.75 mm diameter filaments suitable for FFF printing. A single-screw extruder with a custom filament die was used (Collin E 12 P, Maitenbeth, Germany). The extrusion temperature was set to ~250 °C (above PA11’s normal melt temperature to account for the presence of fibers). Filament winding speed and cooling were adjusted to achieve a consistent diameter of 1.75 ± 0.05 mm

Injection Molding: For baseline comparisons, standard tensile bars were injection-molded from each compound (including 35 wt%). An Arburg Allrounder 320C injection molding machine, (Arburg, Lossburg, Germany) was used to mold ISO 527-2 [33] Type 1A dogbone specimens. The barrel temperature was ~240–250 °C and mold temperature ~80 °C, optimized for PA11. Molding was performed immediately after compounding (with the compound re-dried before molding to remove any moisture reabsorption). Each fiber-filled PA11 grade yielded ~17–19 good tensile specimens per batch which have been tested. Unreinforced PA11 specimens were not molded (neat PA11 properties were taken from the supplier’s datasheet for reference). Additionally, a reference short fiber material (PA12 with 4 wt% CF) was injection-molded under similar conditions to serve as a “conventional” composite baseline. This PA12-CF4% corresponds to a typical minor reinforcement used industrially, allowing contrast with the higher fiber content PA11 composites.

Additive Fusion Technology (AFT) Printing: Using the produced filaments, plates were printed with the 9T Labs AFT system (Red Series^®^ machine, 9Tlabs, Zürich, Switzerland). The printing was performed in planar 0° orientation, meaning all filament toolpaths were laid parallel to one another in each layer (aligned with the plate’s length). The filament was deposited through a 0.6 mm diameter nozzle at ~250 °C onto a heated build platform. Each plate was built to dimensions of approximately 100 × 100 mm in-plane, with a total thickness of 4 mm (achieved by ~20 layers of 0.2 mm height each, printed one atop another in the same 0° direction). This raster strategy produces a pseudo-UD (unidirectional) composite, with fibers highly aligned along one axis. During extrusion, the carbon fibers were strongly oriented in the flow direction by the shear in the nozzle, so the printed roads contained fibers predominantly along the 0° print direction. After printing, the plates contained some void between roads and layers (initial void content estimated at 10–20% by volume, typical for FFF with carbon fiber). To minimize porosity and fuse the layers, each printed plate was then consolidated. Consolidation was performed by placing the plate in a hot press at 200 °C under ~2 MPa pressure for 5 min, then cooling under pressure. This collapsed most voids, achieving a final porosity below 1%. The resulting consolidated plates were flat, fully dense composite laminates with short fibers oriented along the in-plane longitudinal direction.

Specimen Extraction: From each consolidated plate, test specimens were machined. For tensile testing of AFT samples, rectangular flat bar specimens (dimensions ~80 × 10 × 4 mm) were CNC-milled. Two principal orientations were prepared: 0° specimens, with their length aligned to the printing fiber direction (to test strength/stiffness along fibers), and 90° specimens, with length perpendicular to fiber alignment (to test properties transverse to fibers). Only one plate per fiber content (15% and 25%) was printed, so to obtain multiple specimens, the plate was partitioned accordingly. For each fiber content we obtained ~5 tensile specimens at 0°, ~5 at 90°, plus a few smaller specimens reserved for impact and DMA. The injection-molded dogbones were tested in the as-molded orientation (fiber alignment naturally followed the dogbone axis due to flow). All tests have been performed in dry conditions related to the standard recommended drying process of 4 h at 80 °C.

### 2.3. Fiber Length Distribution Measurement

Fiber length distributions after processing were determined by dissolving/burning off the matrix and measuring the residual fiber lengths. Small samples of each compound (before filament extrusion) were subjected to matrix removal using a muffle furnace: ~2 g of each composite was placed in a ceramic crucible and heated to ~600 °C until all PA11 was ashed away (with careful control to avoid fiber oxidation). The remaining carbon fibers were collected and dispersed in water with a surfactant. Using optical microscopy and image analysis, over 500 fibers per sample were measured to build a statistical length distribution. A software tool (FiberApp (Version 2016-10-12), ETH Zürich, Switzerland) was calibrated with the image scale to convert pixel lengths to millimeters. Two replicate measurements were performed for each fiber loading to ensure reliability. Because some fine fiber fragments (dust) may volatilize or clump during ashing, the measured distributions are considered semi-quantitative (trends rather than absolute). Additionally, the fiber mass fraction in each compound was verified by comparing initial and residual weights (thermogravimetric analysis was considered for precision, but a wet chemical digestion was ultimately used for low fiber contents since TGA (TA Instruments, New Castle, DE, USA) was less accurate at such low residue levels).

### 2.4. Mechanical Testing

Quasi-static tensile tests were performed according to ISO 527-1 [34]. For the injection-molded specimens (Type 1A dogbones), a gauge length of 50 mm and a width of 10 mm was used. For the AFT-printed flat specimens, a similar gauge length (~50 mm) was marked on the 80 mm total length, and the width was 10 mm; the thickness was the full 4 mm plate thickness. Tests were carried out on a Zwick universal testing machine with a 100 kN load cell (Zwick Roell, Germany). A strain rate corresponding to 1 mm/min crosshead speed was used to determine tensile modulus, followed by 5 mm/min for the remainder to failure. An extensometer measurement tracked axial strain in the gauge. At least five specimens per condition were tested (for AFT 0° and 90° at each fiber content, and for each injection material). The measured properties reported are tensile strength, Young’s modulus, and strain at break.

Charpy Impact Testing: Notched Charpy impact tests were conducted following ISO 179-1 [35]. From each material, unnotched rectangular bars (80 × 10 × 4 mm) were prepared. The tests used a pendulum impact tester with a 4 J hammer, measuring the energy absorbed in breaking the specimen (which was supported as a simply supported beam in the impact apparatus). At least 3–5 specimens per variant were tested. For the printed material, both 0° and 90° orientations were tested (meaning the notch was always perpendicular to the specimen length, so for 0° specimens the notch plane was parallel to fiber direction, whereas for 90° specimens the notch plane cut across fibers aligned along the specimen width).

Dynamic Mechanical Analysis (DMA): DMA tests were performed in three-point bending mode on a TA Instruments Q800 DMA (TA Instruments, New Castle, DE, USA). Small beam specimens (~35 × 10 × 4 mm) were cut from the composites. Two specimens from each AFT plate (one aligned 0°, one 90°) and from injection-molded material were tested. The support span was 15 mm. Tests were run in multi-frequency strain-controlled mode: temperature was ramped from 25 °C to 130 °C at 2 °C/min, with dynamic flexural strain within the linear viscoelastic range. Frequencies of 1 Hz (and an additional 10 Hz, 100 Hz for one set) were applied to observe any frequency dependence. The key data obtained were the storage modulus (E′) as a function of temperature and the glass transition temperature. Since PA11 is semi-crystalline (melting point ~186 °C) and has a moderate glass transition around 45–50 °C, the temperature sweep to 130 °C covered the glass transition (Tg) region and part of the rubbery plateau but stayed well below melting.

Microstructure and Fiber Orientation: To evaluate fiber orientation in the printed vs. molded samples, optical microscopy was performed on polished cross-sections. Specimens were cut from the composites and potted in epoxy resin. For AFT samples, sections were prepared both parallel to the print direction and perpendicular to it. The surfaces were ground and polished (down to 1 µm diamond paste) to reveal fiber cross-sections. A Keyence digital microscope at 200–300× was used to image the microstructure.

## 3. Results

It was found that the maximum fiber content for successful filament extrusion was ~25 wt%. The 35 wt% compound led to frequent filament breakage and clogging due to its high viscosity and brittleness. Material with 35 wt% fibers could not be continuously extruded into filament, confirming a practical upper limit around 30 wt% fiber for this process. Therefore, only the 15 wt% and 25 wt% filaments were produced and used for printing, while the 35 wt% compound was characterized via injection molding only.

### 3.1. Fiber Length Distribution After Processing

**Figure 2:**
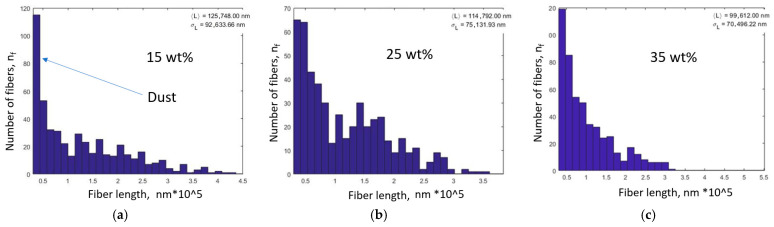
Fiber length distribution in PA11/CF composites after compounding, for (**a**) 15, (**b**) 25, and (**c**) 35 wt% fiber loadings. Data from ~500 fibers per sample.

The compounding process led to a drastic reduction in fiber length for all composites. The original 6 mm fibers were shortened to sub-millimeter lengths. The measured fiber length distributions for 15 wt%, 25 wt%, and 35 wt% fiber content are summarized in Figure 2. The distributions were approximately log-normal, spanning from very fine “dust” fibers (~0.02 mm) up to a few longer remnants (~0.3–0.4 mm). The average fiber length decreased with increasing fiber content (Figure 2a–c). At 15 wt% CF, the mean fiber length was about 0.12 mm, at 25 wt% ~0.11 mm, and at 35 wt% ~0.09 mm. In other words, the fibers retained only ~2% of their initial length on average by the time the compound was processed. This trend is attributed to more intensive fiber–fiber interactions at higher loadings, causing greater attrition during mixing. A linear fit to the data in Figure 2a–c extrapolates that further increases in fiber fraction would continue to shorten the fibers, eventually approaching a critical length below which reinforcement efficiency diminishes significantly.

We report standard deviations (~0.07, 0.06, 0.05 mm for 15, 25, 35 wt%) and 95% confidence intervals for the means. A linear regression of mean length versus wt% shows R^2^ ≈ 0.96 (*p* ≈ 0.12; limited by three data points). Measurement uncertainty arises from ashing-induced fragmentation and image analysis resolution, addressed by duplicate measurements and ~500 fibers/sample.

Most fiber attrition occurred during the kneading step. Filament extrusion introduced some additional shear, but the majority of breakage had already occurred. The presence of fine pulverized fiber fragments (“dust”) increased with fiber loading, evidenced by a higher proportion of very short fibers in the 35% sample. Some of this dust may even be lost during processing or analysis, meaning the actual fiber length distribution could be skewed toward shorter lengths than measured.

These results underscore a key challenge that while adding more fibers generally increases stiffness, it also accelerates fiber breakage, reducing aspect ratio. Beyond a certain fiber content (~25–30 wt% in this material system), the incremental gains in stiffness and strength are expected to diminish with the reduction in fiber aspect ratio.

### 3.2. Tensile Properties

**Figure 3:**
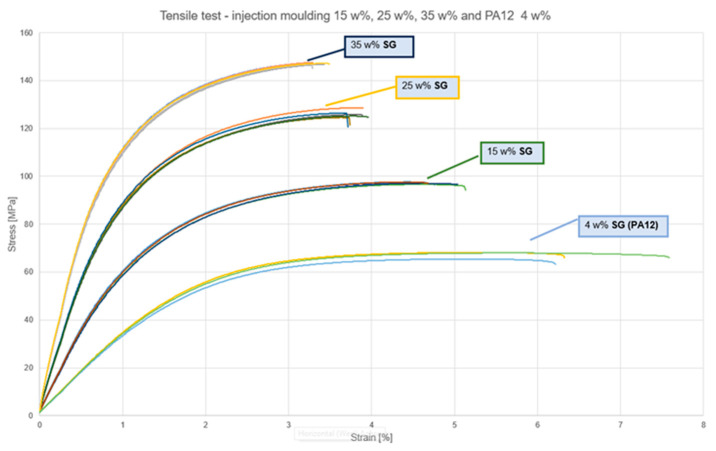
Tensile test—injection molding 15 wt%, 25 wt%, 35 wt% and PA12 4 wt%. The curves within each group (shown in different colors) correspond to repetitions of the test on the same sample type.

**Figure 4:**
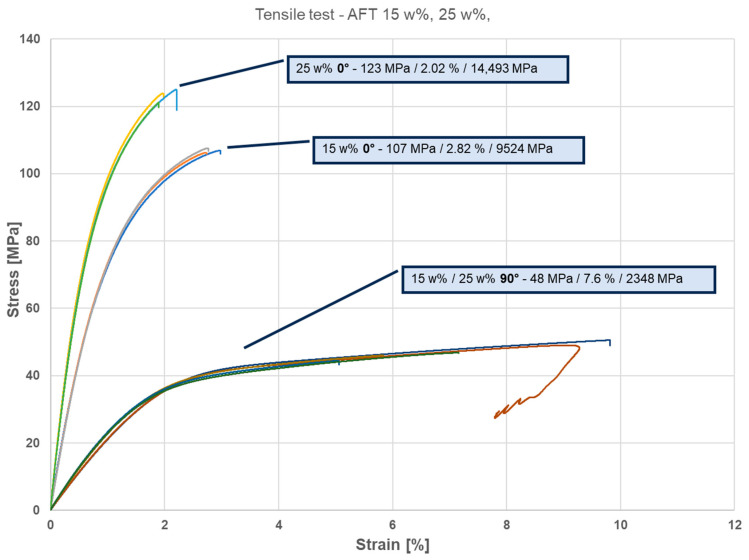
Tensile test—3D printed (AFT) 15 wt%, 25 wt %, different directions. The curves within each group (shown in different colors) correspond to repetitions of the test on the same sample type.

**Figure 5:**
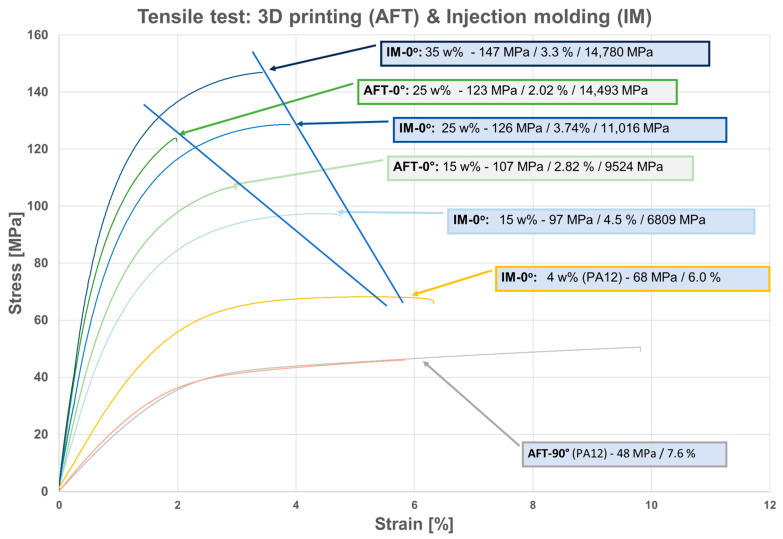
Tensile test—3D printed (AFT) and Injection molding (IM) comparison.

The tensile test results (stress-strain) for both injection-molded (IM) and AFT-printed composites are shown in Figure 3 and Figure 4, respectively. Additionally, Table 1 lists the numerical values for tensile strength and Young’s modulus for different samples. Figure 5 groups the experimental results for comparison between injection-molded samples and AFT ones. Injection-molded PA11/CF samples displayed the expected increase in tensile strength and modulus with fiber content. Axial tensile strength rose from 97 MPa at 15 wt% CF to 126 MPa at 25 wt% and 147 MPa at 35 wt%, while Young’s modulus increased from 6.8 to 11.0 to 14.8 GPa over the same range. The strain at break decreased modestly with fiber content, from 4.5% down to 3.3%. Gains from 25% to 35% were less pronounced, indicating some saturation as fiber length decreased and dispersion became more challenging.

In the 0° direction, tensile strengths were 107 MPa at 15 wt% and 123 MPa at 25 wt%—comparable to, or slightly higher than, the injection-molded samples. At 15% fiber, the AFT 0° strength (~107 MPa) exceeded IM 15% (~97 MPa), attributed to better fiber alignment. At 25% fiber, the AFT 0° strength (~123 MPa) matched IM 25% (~126 MPa). The tensile modulus for AFT 0° samples was significantly higher than for IM: 9.5 GPa vs. 6.8 GPa at 15%, and 14.5 vs. 11.0 GPa at 25%. These correspond to ~40% and ~32% higher stiffness, respectively. The trade-off was a lower elongation at break for AFT 0° (2.0–2.8%) compared to IM (3.3–4.5%). Another observation in Figure 4 is that the AFT-printed composites exhibited strong anisotropy, and there is little difference between the responses of the 90° samples with 15 and 25 wt% CF.

In contrast, the AFT 90° specimens (transverse load) showed very low strength and stiffness. Their tensile strength was only ~50 MPa at 15% CF and ~46 MPa at 25% CF, compare to the strength of neat PA11 (~37 MPa) with only a slight contribution from fibers. The tensile modulus was ~2.1–2.6 GPa, only ~20% of the 0° modulus and even below typical neat PA11 (~1.2 GPa). However, the 90° specimens displayed high ductility: the 15% ° sample reached ~9.2% strain and the 25% 90° sample ~6%, approaching neat PA11 levels.

In general, injection-molded composites showed a ductile polymer composite response with yielding at ~3–4% strain and some post-yield deformation before fracture. Increasing fiber content elevated strength and modulus but reduced elongation. AFT 0° specimens displayed steep elastic regions and abrupt brittle failure at low strain, while AFT 90° specimens exhibited very low modulus and long yielding plateaus similar to neat PA11. AFT maximized axial properties, with 25% CF AFT 0° samples achieving ~32% higher modulus than injection-molded equivalents. Strength was similar between the two processes at the same fiber fraction, but ductility was much lower for AFT.

### 3.3. Impact Strength (Charpy)

**Figure 6:**
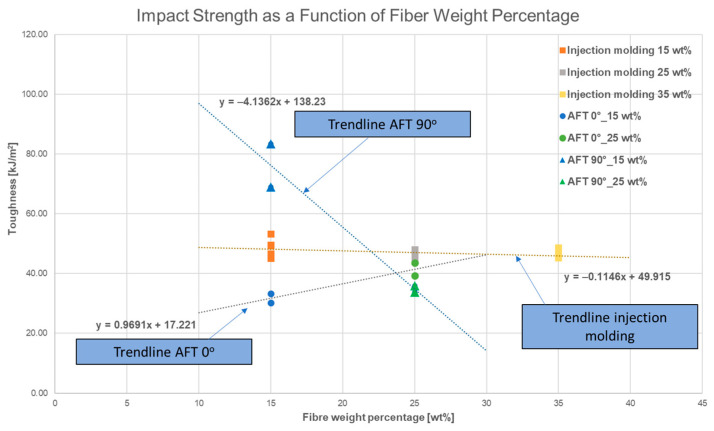
Impact Strength as a Function of Fiber Weight Percentage.

Charpy impact tests revealed clear trends in toughness which can be observed in Figure 6. Injection-molded PA11/CF maintained stable impact strength (~46–49 kJ/m^2^) across 15–35 wt% CF, indicating that higher fiber content did not significantly embrittle the composites. The random fiber orientation preserved toughness even at higher loadings.

AFT composites showed strong orientation effects. The 0° orientation had lower impact strength than IM, with 31.8 kJ/m^2^ at 15% CF (about 35% lower than IM15) and 41.5 kJ/m^2^ at 25% CF (narrowing the gap to IM25). The 90° orientation at 15% CF had the highest toughness of all (~76 kJ/m^2^, 55% higher than IM15), as the matrix dominated failure and fibers acted as crack deflectors. However, at 25% CF, 90° toughness dropped drastically to ~34.8 kJ/m^2^ due to delamination and crack propagation along fibers. The numerical values are summarized in Table 2.

In summary, AFT can either improve or reduce toughness depending on orientation and fiber content. Quasi-isotropic layups or lower fiber fractions may mitigate brittleness.

### 3.4. Dynamic Mechanical Analysis (DMA)

**Figure 7:**
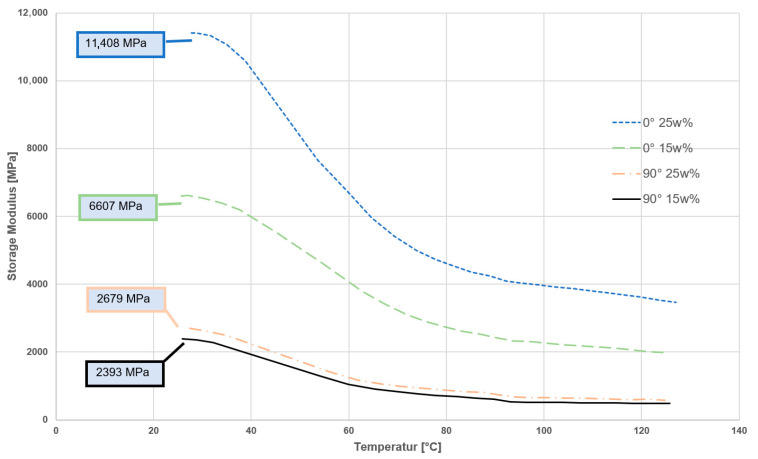
DMA test—3D printed (AFT) 15 wt%, 25 wt%, different directions.

DMA confirmed the temperature-dependent stiffness differences. At 30 °C, AFT 0° composites had the highest storage modulus (~11.4 GPa at 25% CF) and AFT 90° the lowest (~2–3 GPa). As the temperature approached the PA11 glass transition (~45 °C), all samples showed reduced modulus (Figure 7).

### 3.5. Microstructure and Fiber Orientation Analysis

**Figure 8:**
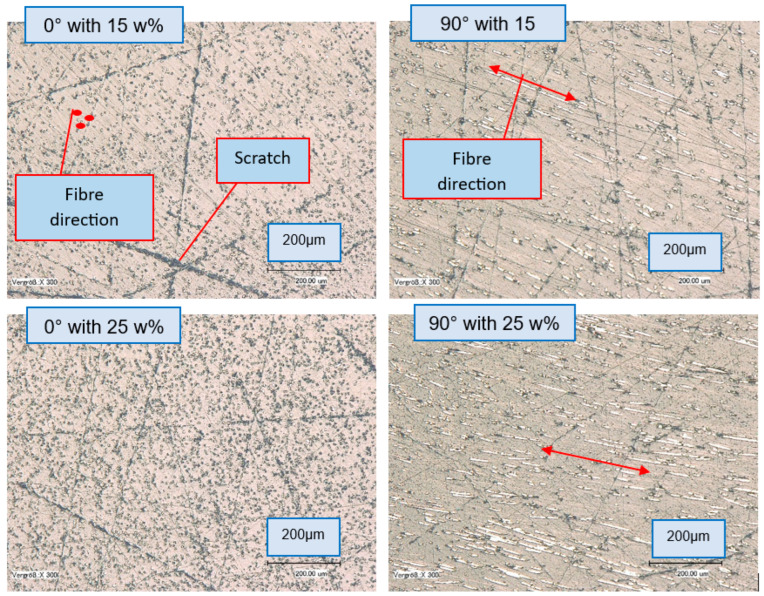
Microsection images—3D printed (AFT) 15 wt%, 25 wt%, different directions.

Micrographs confirmed differences in fiber orientation of samples produced with two manufacturing methods. As can be seen in Figure 8, AFT samples displayed almost fully unidirectional fibers, with 0° sections showing mainly fiber cross-sections (dots) and 90° sections showing elongated fibers along the print direction. Injection-molded samples typically show mixed orientations, with some flow-aligned fibers at the edges and more random orientation in the core (skin–core effect).

Porosity was minimal in consolidated AFT, so property differences were due to fiber orientation and length rather than voids.

In printed plates, two orientations were observed: a cross-section parallel to print direction (showing fibers mostly end-on as dots) and one perpendicular (showing fibers longitudinally as lines in-plane). These correspond to the tensile specimen orientations: the 0° specimen cross-section reveals fiber ends (since fibers run along the length), whereas the 90° specimen cross-section reveals fibers lying in the plane. The degree of fiber alignment was assessed qualitatively by the shape of fiber cross-sections: perfectly aligned fibers appear as circular dots when cut crosswise, whereas random or transverse fibers appear as elongated shapes or fiber lengths in-plane. The aim of this paper was not to use the fiber orientation distribution for precise modeling, so µ-Computed Tomography (CT) analysis was not required for a more accurate 3D fiber orientation distribution. However, the current approach has the advantage of inspecting a larger surface for fiber analysis compared to typical CT volumes, which are around 1 × 1 × 3 mm [25].

## 4. Discussion of the Results

The results illustrate the distinct mechanical responses of short fiber composites produced by AFT (additive manufacturing) versus injection molding, and how these relate to differences in microstructure, particularly fiber orientation and fiber length.

**Figure 9:**
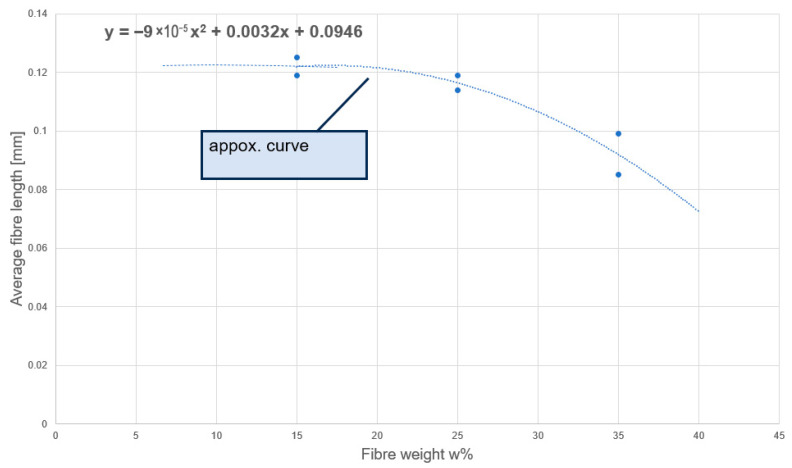
Ratio between Fiber Length Distribution to Fiber Mass Percentage.

Compounding reduced the 6 mm starting fibers to an average length of ~0.1 mm (100 µm) or less, with higher fiber loadings (larger fiber weight percentage) causing greater attrition (mean ~0.085 mm at 35% CF). This severe shortening is common in short fiber compounding. It also suggests that beyond a certain fiber volume fraction, the additional fibers essentially pulverize each other, resulting in a rapid drop in fiber length as content increases. In our case, the data suggested a roughly linear decrease in mean length with fiber wt% (see Figure 9), though at higher fiber loadings this is likely to become nonlinear. The implication is that simply adding more fibers yields diminishing returns. Although fiber fraction increases, the aspect ratio simultaneously decreases, reducing reinforcement efficiency. It should also be noted that at high fiber volume fractions, the melt flow becomes severely restricted due to fiber-fiber interactions, making further increases in fiber content impractical.

The experimental stress-strain curve shows that the jump in tensile modulus from 15% to 25% CF was large (+62% for IM, +52% for AFT 0°), but from 25% to 35% (injection only) the increase was smaller (+34% for modulus, +17% for strength). At some point the composite’s strength may plateau or drop if fiber length falls below the critical length for reinforcement. In practice, our finding that ~30 wt% CF is the maximum processable in filament form aligns with the idea that beyond this level, the drawbacks (difficult extrusion and excessively short fibers) outweigh the benefits.

The most striking differences between AFT and injection samples arise from fiber orientation distribution. Injection molding, due to the complex cavity flow, results in a quasi-random 3D orientation with a mild flow bias. In contrast, AFT printing produced an almost unidirectional in-plane orientation. This made the printed composites behave almost like unidirectional laminates, whereas the injection composites behaved like short fiber mats with partial alignment. The advantages of alignment are evident in the axial tensile behavior as the AFT 0° specimens achieved stiffness and strength approaching the theoretical upper bound for a given fiber content. However, the AFT 0° 25% composite reached 123 MPa tensile strength, very close to the injection 25% sample at 126 MPa.

The anisotropy in AFT parts is both an advantage and a limitation. On one hand, it allows tailoring of properties—fibers can be placed along stress paths to maximize performance. On the other hand, loads perpendicular to fiber orientation find the material weak, as only the matrix carries stress and fibers act as stress concentrators. This, of course, can be mitigated by printing unidirectional layers in different orientations, like those used in multidirectional continuous fiber laminates. Injection-molded materials, with their less oriented reinforcement, avoid such severe directional behavior. In structural terms, AFT parts are orthotropic, requiring careful design consideration, while injection-molded parts are closer to quasi-isotropic.

Toughness was strongly affected by fiber alignment. AFT 0° specimens showed the lowest fracture strain (~2%) and significantly lower Charpy impact energy compared to injection-molded ones. This reflects brittle, fiber-dominated fracture where cracks propagate straight through aligned fibers. Injection molded composites and AFT 90° specimens allowed more matrix deformation and crack tortuosity, resulting in higher toughness. Notably, AFT 90° specimens at 15% CF reached very high impact energy (~76 kJ/m^2^), behaving much like neat PA11. However, at 25% CF, toughness dropped drastically due to delamination and crack propagation along fibers. This may also explain why we did not observe a clear difference in strength between the AFT and injection-molded samples at 25% CF.

Interestingly, injection-molded composites retained toughness even at 35% CF (~46 kJ/m^2^). This highlights PA11’s inherent toughness, which prevents embrittlement even at high fiber loadings. In comparison, PA12 with only 4% CF retained very high toughness, behaving almost like neat PA12. This shows that low fiber fractions or random orientation preserve polymer-like toughness, while aligned high fiber contents compromise it. For AFT prints, improvements may come from multi-directional layups (0/90 or ±45°) or tougher matrices to mitigate anisotropic brittleness.

Consolidation in AFT was essential as-printed parts had visible porosity (10–20%), which would degrade properties. By hot-pressing the printed preforms, porosity was reduced to below 1%, producing properties comparable to injection molding. Our results confirm that consolidation is necessary for high performance, though it adds an extra step. Without it, transverse strength would drop severely as voids combined with fiber alignment to form easy fractured planes. Thus, the two-step AFT process (print then consolidate) is validated as crucial.

It should be noted that Continuous fiber PA11 tapes reach tensile strengths near 1000 MPa and moduli near 60 GPa, far above our short fiber composites (max 147 MPa, 14.8 GPa). Short fibers are both lower in volume fraction and far shorter than the load transfer length, limiting performance. Still, AFT alignment narrows the gap: at 25 wt% CF, AFT 0° matched injection strength and exceeded its stiffness. If higher fiber fractions could be processed into filaments without excessive breakage, performance could rise further, but fiber attrition currently sets a practical limit.

This study demonstrates a sustainable approach by combining a bio-based matrix (PA11) with potential recycled carbon fibers, highlighting that PA11/rCF achieves properties comparable to petroleum-based composites. The AFT process further minimizes scrap compared to traditional lay-up methods. Moreover, parts remain recyclable at end-of-life since both matrix and fibers can be reclaimed, though fiber length will shorten further.

Finally, PA11 proved thermally stable up to ~130 °C and well suited for processing by both injection molding and AFT. This robustness, combined with the potential of recycled carbon fibers, highlights PA11/CF composites as a viable and sustainable choice for lightweight structural applications.

## 5. Conclusions

This study compared short carbon fiber-reinforced PA11 composites manufactured by injection molding (IM) and Additive Fusion Technology (AFT) with consolidation, highlighting the influence of fiber content, fiber orientation, and processing method on performance.

- Filament fabrication and process limits: PA11 was successfully compounded and extruded into 1.75 mm filaments with up to 25 wt% carbon fibers. At 35 wt%, filament breakage and clogging occurred, establishing a practical extrusion limit near 30 wt% due to high viscosity, brittleness, and severe fiber attrition.
- Fiber length reduction: Processing shortened the 6 mm starting fibers to an average near 0.1 mm. Higher fiber contents led to more breakage (~0.12 mm at 15% CF → ~0.09 mm at 35% CF), reducing aspect ratios and limiting reinforcement efficiency. Beyond 25 wt%, performance gains diminish despite adding more fibers.
- Mechanical performance: Injection-molded composites showed steadily increasing strength and stiffness up to 147 MPa and 14.8 GPa at 35 wt% CF, while maintaining good toughness (~46 kJ/m^2^) and moderate ductility (~3–4% strain). AFT composites exhibited strong anisotropy:
  ○ 0° orientation: High strength (123 MPa) and modulus (14.5 GPa) at 25 wt% CF, comparable to injection molding.
  ○ 90° orientation: Very low strength (~46–50 MPa) and modulus (~2.3 GPa), behaving almost like neat PA11.
  ○ AFT 0° samples had >5× higher stiffness than 90° but lower impact toughness, whereas 90° specimens at 15% CF showed high toughness (~76 kJ/m^2^) that dropped sharply at 25% CF due to delamination.
- Microstructure and thermal behavior: Microscopy confirmed unidirectional fiber alignment in AFT prints versus mixed quasi-random orientations in injection moldings, explaining the strong directional dependence of AFT properties. Consolidation reduced porosity below 1%, confirming that property differences arise from fiber orientation and length rather than voids. DMA showed that fiber reinforcement increased the storage modulus in the glassy state, with AFT 0° samples retaining the highest stiffness up to ~80 °C. The glass transition temperature (Tg ≈ 45–50 °C) was unaffected by fiber content or orientation.
- Design and sustainability implications: This study demonstrates that combining a bio-based PA11 matrix with recycled carbon fibers achieves mechanical performance comparable to petroleum-based composites such as glass fiber-reinforced PA6, offering a more sustainable alternative.
  ○ Injection molding is better suited for applications requiring quasi-isotropic properties and high impact resistance.
  ○ AFT printing is ideal when maximum stiffness and strength along a defined load path are critical, but anisotropy must be carefully considered.
  ○ A fiber content of around 25 wt% represents an optimal balance between processability and performance in filament-based AFT.

Future work should explore multi-directional layups, hybrid short/continuous fiber composites, and optimized processing strategies to preserve fiber length and improve interlayer bonding as an example used in [31]. Additionally, incorporating impact modifiers may help mitigate the brittleness of highly aligned AFT composites.

End-of-Life Recyclability of PA11/CF Composites

Matrix Recovery: PA11 is a thermoplastic, which means it can be remelted and reprocessed. At the end-of-life, PA11/CF composites can be mechanically shredded and re-extruded, allowing for partial property retention. However, repeated reprocessing causes chain scission and some loss of toughness and elongation.

Fiber Recovery: The carbon fibers embedded in PA11 can be reclaimed by mechanical, thermal, or chemical recycling routes. Mechanical shredding yields very short, damaged fibers with limited reinforcing potential. Thermal recycling (e.g., pyrolysis) removes the PA11 matrix and recovers fibers with reduced surface quality, while chemical recycling (e.g., solvolysis) can yield cleaner fibers closer to virgin quality.

Closed-Loop Potential: Since both components (bio-based PA11 and recycled carbon fibers) are recyclable, PA11/CF composites support circular economy concepts. Recovered fibers can be compounded into new thermoplastic matrices, while PA11 waste can potentially be re-used or chemically depolymerized.

Limitations: Fiber length attrition accelerates with each cycle, lowering reinforcement efficiency. Matrix degradation (oxidation, chain scission) during thermal or mechanical recycling can reduce overall material performance. Achieving stable, high-value recycling requires optimized re-sizing of fibers and stabilization of the PA11 matrix.

Overall, AFT printing enables short fiber composites with directional performance approaching that of continuous fiber laminates, while injection molding remains advantageous for balanced, impact-tolerant designs. These findings provide design guidelines for lightweight, sustainable structures and confirm PA11/rCF composites as a promising path toward recyclable, high-performance materials.

## Figures and Tables

**Table 1:** Tensile properties of short CF/PA11 composites produced by injection molding (IM) vs. AFT printing (0° and 90° orientations). Values are mean (std. dev.).

Material	Fiber (wt%)	Tensile Strength σ_m_ (MPa)	Young’s Modulus E (GPa)
PA11—IM (15%)	15 wt% CF	97.0 (±0.4)	6.809 (±153)
PA11—IM (25%)	25 wt% CF	126.0 (±1.6)	11.016 (±139)
PA11—IM (35%)	35 wt% CF	147.0 (±0.3)	14.780 (±234)
PA11—AFT 0° (15%)	15 wt% CF	107.0 (±0.7)	9.524 (±72)
PA11—AFT 0° (25%)	25 wt% CF	123.0 (±2.0)	14.493 (±318)
PA11—AFT 90° (15%)	15 wt% CF	50.0 (±1.3)	2.121 (±246)
PA11—AFT 90° (25%)	25 wt% CF	46.0 (±1.2)	2.575 (±46)

**Table 2:** Charpy notched impact strength of PA11/CF composites (average ± st.dev).

Material	Fiber (wt%)	Notched Charpy Impact (kJ/m^2^)
PA11—Injection 15% CF	15%	48.75 ± 3.08
PA11—Injection 25% CF	25%	45.94 ± 1.50
PA11—Injection 35% CF	35%	46.46 ± 1.34
PA11—AFT (0°) 15% CF	15%	31.76 n.a.
PA11—AFT (0°) 25% CF	25%	41.45 n.a.
PA11—AFT (90°) 15% CF	15%	76.18 n.a.
PA11—AFT (90°) 25% CF	25%	34.82 n.a.

## Data Availability

The original contributions presented in this study are included in the article. Further inquiries can be directed to the corresponding authors.
